# BK Channels Mediate Cholinergic Inhibition of High Frequency Cochlear Hair Cells

**DOI:** 10.1371/journal.pone.0013836

**Published:** 2010-11-04

**Authors:** Eric Wersinger, Will J. McLean, Paul A. Fuchs, Sonja J. Pyott

**Affiliations:** 1 Department of Otolaryngology Head and Neck Surgery, Center for Hearing and Balance, and Center for Sensory Biology, Johns Hopkins University School of Medicine, Baltimore, Maryland, United States of America; 2 Department of Biology and Marine Biology, University of North Carolina Wilmington, Wilmington, North Carolina, United States of America; Claremont Colleges, United States of America

## Abstract

**Background:**

Outer hair cells are the specialized sensory cells that empower the mammalian hearing organ, the cochlea, with its remarkable sensitivity and frequency selectivity. Sound-evoked receptor potentials in outer hair cells are shaped by both voltage-gated K^+^ channels that control the membrane potential and also ligand-gated K^+^ channels involved in the cholinergic efferent modulation of the membrane potential. The objectives of this study were to investigate the tonotopic contribution of BK channels to voltage- and ligand-gated currents in mature outer hair cells from the rat cochlea.

**Methodology/Principal:**

Findings In this work we used patch clamp electrophysiology and immunofluorescence in tonotopically defined segments of the rat cochlea to determine the contribution of BK channels to voltage- and ligand-gated currents in outer hair cells. Although voltage and ligand-gated currents have been investigated previously in hair cells from the rat cochlea, little is known about their tonotopic distribution or potential contribution to efferent inhibition. We found that apical (low frequency) outer hair cells had no BK channel immunoreactivity and little or no BK current. In marked contrast, basal (high frequency) outer hair cells had abundant BK channel immunoreactivity and BK currents contributed significantly to both voltage-gated and ACh-evoked K^+^ currents.

**Conclusions/Significance:**

Our findings suggest that basal (high frequency) outer hair cells may employ an alternative mechanism of efferent inhibition mediated by BK channels instead of SK2 channels. Thus, efferent synapses may use different mechanisms of action both developmentally and tonotopically to support high frequency audition. High frequency audition has required various functional specializations of the mammalian cochlea, and as shown in our work, may include the utilization of BK channels at efferent synapses. This mechanism of efferent inhibition may be related to the unique acetylcholine receptors that have evolved in mammalian hair cells compared to those of other vertebrates.

## Introduction

Outer hair cells (OHCs) are the specialized sensory cells that endow the mammalian cochlea with its extraordinary sensitivity and exquisite frequency selectivity [1], [2]. Cochlear amplification is mediated at least in part by electromotile changes in the length of OHCs in response to sound-evoked receptor potentials [3]. OHC receptor potentials are shaped by both voltage- and ligand-gated ion channels, especially K^+^ channels [4], [5], [6]. Voltage-gated K^+^ channels control the membrane potential directly [4], whereas ligand-gated K^+^ channels, specifically involved in the efferent regulation of the OHC membrane potential, do so indirectly via cholinergic activation of the Ca^2+^ permeable α9α10-containing nicotinic cholinergic receptors (nAChRs) [7], [8] that, in turn, activate Ca^2+^-dependent SK2 K^+^ channels [6], [9] to hyperpolarize and inhibit the OHC.

Although KCNQ4 channels have been implicated as the predominant K^+^ current in mouse OHCs [4], [10], [11], recent studies examining transgenic knockout mice also have implied a role for BK K^+^ channels in high frequency hearing loss [12], [13]. In line with these observations, Engel and others reported a gradient of BK channel immunoreactivity in OHCs that increases from apical (low frequency) to basal (high frequency) turns developmentally [14]. A similar developmental and tonotopic gradient of BK channel expression was reported by Langer and others using *in situ* hybridization [15]. However, previous electrophysiological evidence for the expression of BK channels in OHCs has been much less clear. Mammano and Ashmore recorded from undissociated OHCs from adult guinea pig and reported the expression of distinct K^+^ channels in OHCs from apical turns and basal turns but found no evidence for expression of BK currents in OHCs from either region [16]. In contrast, Housley and Ashmore reported a BK-like current (TEA- and Ca^2+^-sensitive), in whole cell recordings from OHCs isolated from all turns of the guinea pig cochlea [17]. Differences in species and technique could account for the discrepancy in findings.

BK channels are both voltage- and ligand-gated [18], [19], [20] and could contribute to both types of conductances in OHCs. The objectives of this study were to investigate the tonotopic contribution of BK channels to these currents in mature OHCs using both whole-cell patch-clamp recordings and immunofluorescence. We voltage-clamped OHCs in apical (low frequency) and basal (high frequency) segments of the rat cochlea and used specific blockers to determine the contribution of BK channels to voltage- and ligand-gated currents. Quantitative immunofluorescence further defined the expression pattern of BK channels relative to SK channels and efferent innervation. We found that apical OHCs had no BK channel immunoreactivity and little or no BK current. In marked contrast, BK channels contributed significantly to both voltage-gated and ACh-evoked K^+^ currents in basal OHCs, corresponding with prominent BK channel immunolabeling. This work illustrates a novel mechanism of cholinergic inhibition mediated by BK channels that is uniquely employed by OHCs mediating higher frequency hearing.

## Results

### Properties of outer hair cells vary along the tonotopic length of the rat cochlea

**Figure 1:**
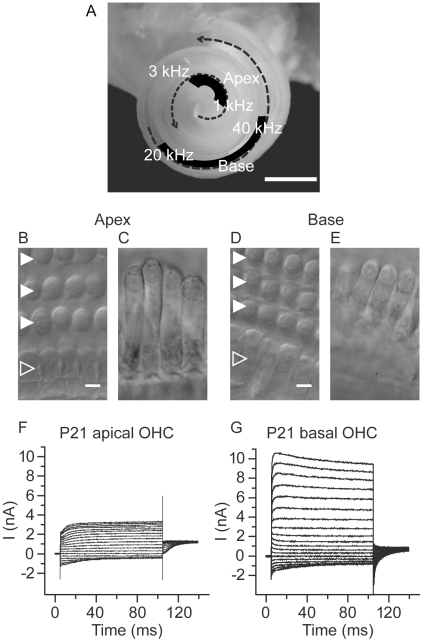
Tonotopic differences in outer hair cell properties. (A) Micrograph showing a P21 rat cochlea in which the bony covering has been removed to expose the intact organ of Corti. The overlayed dashed arrows indicate the progression from low frequency apical turns to higher frequency basal turns. The locations of the apical and basal regions of the organ of Corti (and their approximate frequency ranges) that were examined in this investigation are indicated on black. DIC micrographs of isolated whole mount preparations of apical (B) and basal (D) regions of the organ of Corti show the sensory hair cells organized into a single row of inner hair cells (open arrowhead) and three rows of outer hair cells (solid arrowheads). DIC micrographs of horizontally oriented outer hair cells from apical (C) and basal (E) regions reveal the typical decrease in outer hair cell length from apical (∼50 µm) to basal regions (∼20 µm). Representative records of currents evoked by a series of voltage steps (100 ms in duration) from −131 mV to 49 mV in 10 mV increments (with an interpulse holding potential of −81 mV) show an increase in outward current (at 49 mV) from 2.08±0.09 nA in outer hair cells from apical regions (n = 25, F) to 7.24±0.4 nA in outer hair cells from basal regions (n = 23 basal, G). All scale bars equal 10 µm.

Whole-cell patch-clamp recordings were used to investigate the contribution of BK channels to the membrane conductances of OHCs from apical (low frequency) and basal (high frequency) regions of the cochlea from hearing rats. Following the tonotopic map of the rat cochlea [21], apical regions spanned 85 to 92% (or ∼7.9 to 8.55 mm) of the basilar membrane length (9.3 mm) from the basal tip and corresponded to frequencies between 1 and 3 kHz. Basal regions spanned 25 to 35% (or ∼2.3 to 3.25 mm) of the basilar membrane length and corresponded to frequencies between 20 and 40 kHz (Figure 1A). OHCs from these two regions differed in their overall size, passive membrane properties, and voltage-activated currents similar to previous reports from the guinea pig cochlea [17] and are summarized in Table 1. Although basal OHCs were smaller in size than apical OHCs, they displayed approximately 5-fold greater current density than apical OHCs (Figure 1F–G). Additionally, outward currents activated more rapidly in basal than in apical OHCs (Table 1), and showed varying degrees of inactivation. Outward currents in apical OHCs did not inactivate during the 100 ms step protocol. OHCs from middle turns had outward currents that were larger than those of apical OHCs, but slower than those of basal OHCs (data not shown).

**Table 1:** Properties of outer hair cells recorded in apical and basal regions of the cochlea.

Region	Relative basilar membrane length (%)	Relative basilar membrane length (mm)	Approximate frequency range (kHz)	Size (µm)	C_M_ (pF)	I_Max_ (nA)	τ_Activation_	
							τ_Fast_	τ_Slow_
Apex (n = 25)	85 to 92	7.9 to 8.6	1 to 3	46.6±1.8*	20.5±0.5	2.08±0.09	0.24±0.03	8.9±0.7
Base (n = 23)	25 to 35	2.3 to 3.3	20 to 40	18.7±0.4#	12.7±0.	7.24±0.40	0.18±0.01	2.6±0.4

*n = 3.

# n = 5.

### BK channels do not contribute to voltage-gated potassium currents in apical outer hair cells

**Figure 2:**
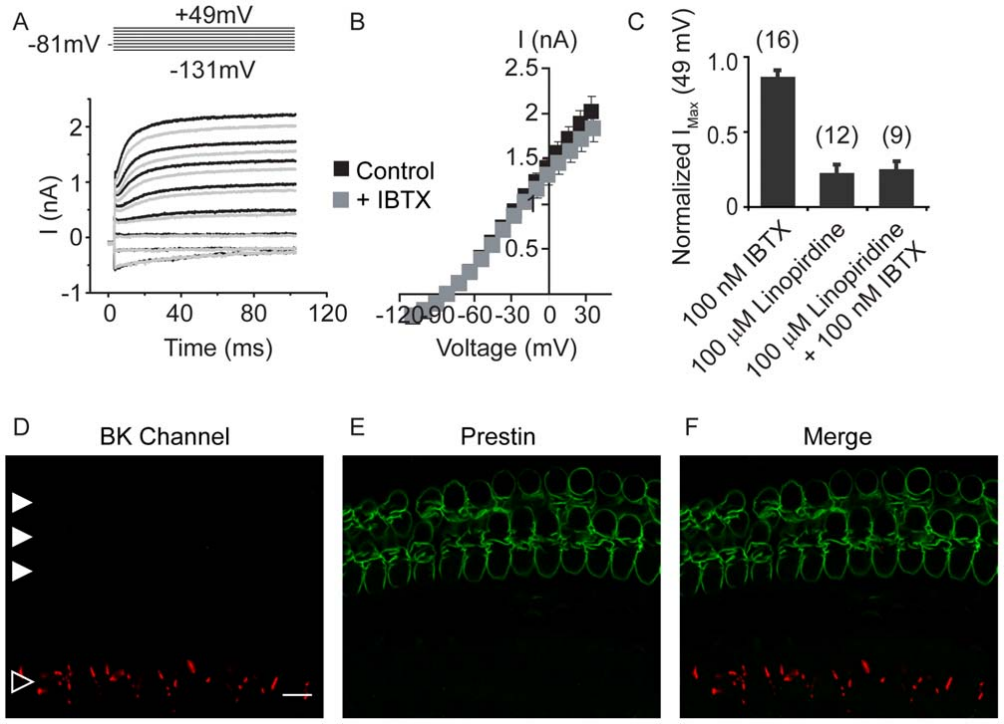
BK channels do not contribute to voltage-gated K^+^ currents in apical outer hair cells. (A) Whole-cell currents recorded from an apical outer hair cell in response to voltage steps (100 ms in duration) from −131 mV to 49 mV in 10 mV increments (with an interpulse holding potential of −81 mV) before (black line) and in the presence (grey line) of bath application of 100 nM IBTX, a specific blocker of BK channels, show little IBTX-sensitive currents. (For clarity only some of the traces are shown.) (B) No differences in voltage-gated currents were observed in response to IBTX application across all test potentials examined in apical outer hair cells. (C) Bar plot comparing the fractional contribution of IBTX- and linopirdine-sensitive currents to apical outer hair cells at 49 mV show that apical outer hair cells express predominantly linopirdine-sensitive KCNQ4 currents and little or no BK currents. Confocal micrograph (1 optical section) of an apical cochlear turn immunostained with a monoclonal antibody against the BK channel (red, D and F) shows immunoreactivity in the region corresponding to the neck of the inner hair cells (indicated with an open arrowhead) but no immunoreactivity in the region associated with the outer hair cells (indicated by the solid arrowheads), immunostained with a polyclonal antibody against prestin (green, E and F). Scale bar equals 10 µm.

Although the functional role of BK channels in inner hair cells (IHCs) is not fully understood, a variety of evidence suggests that they are responsible for the rapidly-activating K^+^ current (IK,f) observed in these cells [12], [22], [23], [24], [25]. Patch clamp recordings of OHCs are technically more difficult, and the contribution of BK channels to OHC membrane conductance is much less described and controversial [26], [27]. Thus, voltage-gated membrane currents were exposed to specific K^+^ channel blockers. Whole-cell voltage-clamp recordings from apical OHCs revealed a family of inward and outward currents in response to depolarization from −131 mV to 49 mV in 10 mV increments (Figure 2A). Application of 100 nM IBTX (a dose that completely blocked BK currents in IHCs) had little effect on activation kinetics (Figure 2A) or the steady state currents (Figure 2B). I_max_ (at 49 mV) was reduced 13.1±2.7% (n = 16) from the control value (Figure 2C). Application of 10 µM paxilline, another BK channel blocker [28], was no more effective than IBTX with a reduction in I_max_ of only 8.7±2.2% (n = 10, p = 0.08) from the control value (Figure S1A).

As reported in apical mouse OHCs [4], [10], outward currents (at 49 mV) were reduced 77.29±3.18% from control (n = 12, p<0.0001) by 100 to 200 µM linopirdine, a KCNQ channel blocker [29] and by 75.74±3.13%, (n = 6, p<0.0001; Figure 2C) from control in 100 µM XE991 (a linopirdine analog; Figure S1A). These findings suggest that outward K^+^ currents in apical OHCs from the rat cochlea flow largely through KCNQ-type K^+^ channels. Importantly, the combined application of linopirdine and IBTX was no more effective than linopirdine alone at reducing outward currents (74.73±3.25%, n = 9, p = 0.2704; Figure 2C).

Although BK channels in IHCs appear to gate independently of intracellular [Ca^2+^] [25], it is possible that BK channels expressed in OHCs may be more similar to the BK channels found in other cells, which are synergistically activated by membrane depolarization and increases in intracellular [Ca^2+^] [30]. Since mature OHCs have quite small voltage-gated Ca^2+^ currents [31] and the foregoing recordings were conducted with relatively strong Ca^2+^ buffering (5 mM EGTA, free Ca^2+^ calculated to be ∼4 nM), it is possible that BK channels were present but unable to activate in the presence of minimal intracellular Ca^2+^. Indeed, even long voltage steps (2.5 to 3 s) were not sufficient to activate the high affinity Ca^2+^-dependent SK2 channels present in OHCs (data not shown) as previously reported for immature IHCs [9].

Therefore, we tested for the presence of BK currents with potentially low affinity gating in apical OHCs with elevated external Ca^2+^ (10 mM) and weak cytoplasmic buffering (0.1 mM EGTA) as well as a preceding depolarization to −20 mV (500 ms) to increase cytoplasmic [Ca^2+^]. In these conditions, Imax (at 49 mV) slightly increased from 151.45±9.15****pA/pF (n = 8) in the control condition to 181.87±14.94 pA/pF in elevated Ca^2+^ (n = 8 OHCs, p = 0.2381), with no apparent change in the activation kinetics. Importantly, Imax was not significantly reduced by the application of 100 nM IBTX (164.10±19.23 pA/pF, n = 8, p = 0.0603; Figure S1B and C). These findings suggest that BK channels are not functional in apical OHCs.

To detect BK channels in apical OHCs, we immunolabeled cochlear segments using a monoclonal antibody directed against the C-terminus of the pore-forming α subunit (Figure S2) and examined them in confocal z-stacks. As described previously [24], [32], the BK channel antibody consistently labeled the neck of IHCs (Figure 2D). However, virtually no BK immunoreactivity was associated with prestin-labeled OHCs in apical cochlear turns (Figure 2E and F).

### BK channels contribute to voltage-gated potassium currents in basal outer hair cells

**Figure 3:**
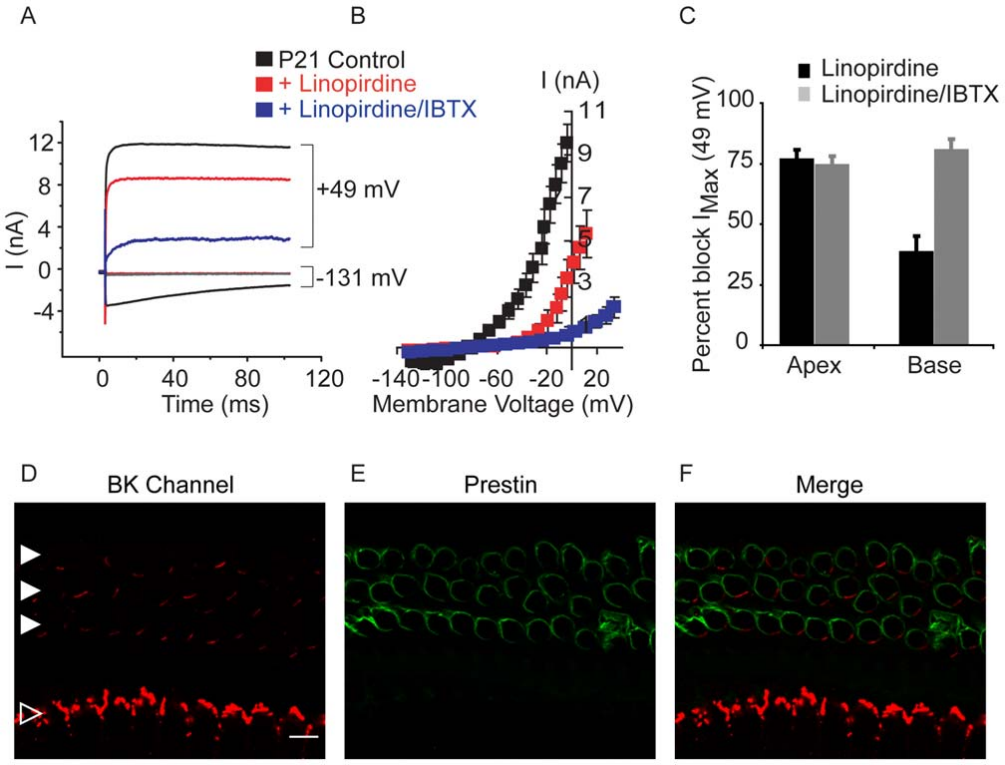
KCNQ4 and BK channels contribute to voltage-gated K^+^ currents in basal outer hair cells. (A) Whole-cell currents recorded from a basal outer hair cell in response to voltage steps (100 ms in duration) from −131 mV to 49 mV in 10 mV increments (with an interpulse holding potential of −81 mV) before (black line) and in the presence of either 100 µM linopirdine (red trace) or both 100 µM linopirdine and 100 nM IBTX (blue trace). (For clarity only 2 potentials, −131 mV and 49 mV are shown.) (B) At depolarizing membrane potentials, both linopirdine-sensitive KCNQ4 and IBTX-sensitive currents contribute to the voltage gated currents in basal outer hair cells. (C) Bar plot comparing the percent block of I_Max_ at 49 mV in apical and basal OHCs in the presence of 100 µM linopirdine (black bars) or both 100 µM linopirdine and 100 nM IBTX (grey bars). (D) Confocal micrograph (1 optical section) of a basal cochlear turn immunostained with a monoclonal antibody against the BK channel (red, D and F) shows immunoreactivity in the region corresponding to the neck of the inner hair cells (indicated with an open arrowhead) as well as immunoreactivity in the outer hair cells (indicated by the solid arrowheads), immunostained with a polyclonal antibody against prestin (green, E and F). Scale bar equals 10 µm.

Despite their smaller size, basal OHCs had substantially larger and faster voltage-gated currents (Figure 3A) than did apical OHCs (Figure 3C). Application of 100 µM linopirdine eliminated the inward currents measured at −131 mV, and partially blocked the outward currents measured at +49 mV (44.72±6.26% reduction, n = 4, p = 0.001; Figure 3A–C), consistent with flow through KCNQ4 channels. Following the application of linopirdine, 100 nM IBTX blocked most of the residual linopirdine-insensitive current (64.47±4.71%, n = 4, p = 0.0121) for a total reduction of the current in the presence of linopirdine and IBTX of 80.93±4.16% (n = 4; Figure 3A–C). In summary, basal OHCs had a greater current density than apical OHCs (Figure 3C). Moreover, while linopirdine-sensitive KCNQ4 currents mediated most of the voltage-gated current in apical OHCs (77.3±3.2%, n = 12), both linopirdine-sensitive KCNQ4 channels and IBTX-sensitive BK channels contributed to voltage-gated currents in basal OHCs (Figure 3C).

Immunofluorescent labeling confirmed the presence of BK channels in basal OHCs (Figure 3D–F). BK channel immunoreactivity in basal OHCs (Figure 3D) was contiguous with the OHC membrane marker prestin (Figure 3E and F). BK immunolabel was restricted to synaptic pole and did not overlap with prestin immunolabel, consistent with prestin's known enrichment in the lateral walls and absence from the synaptic pole [33]. Interestingly, we also consistently observed more BK channel immunoreactivity in basal compared to apical IHCs (Figure 3F compared to Figure 2F). Although this observation was not quantified, it suggests a tonotoptic increase in BK channel expression in IHCs.

### BK channels are associated with efferent terminals in basal outer hair cells

**Figure 4:**
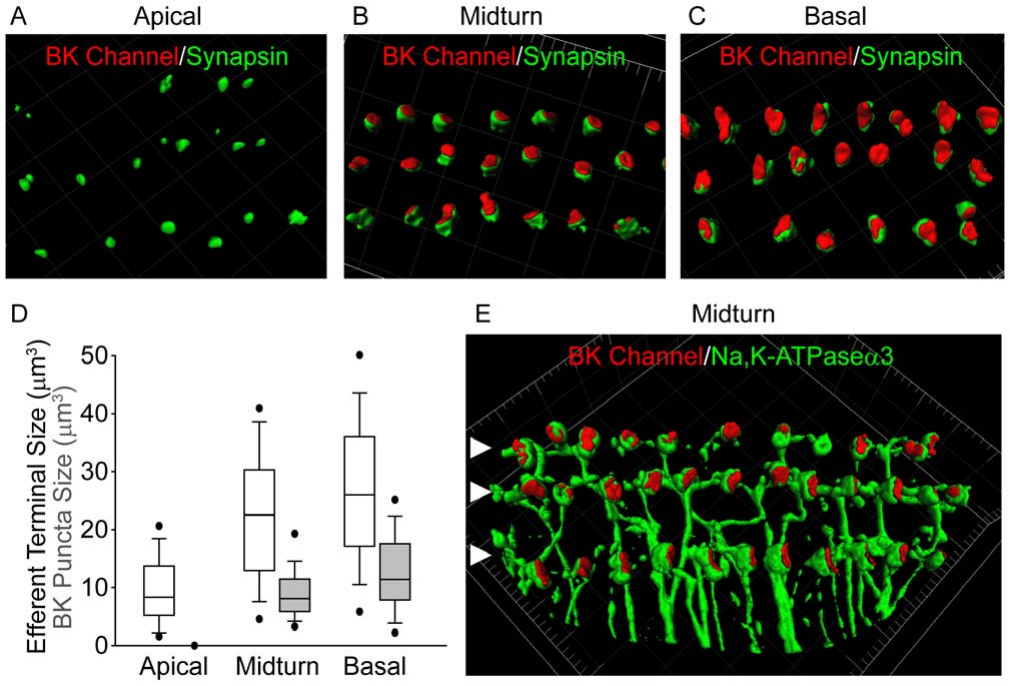
Tonotopic distribution of BK channels in outer hair cells. 3D renderings of confocal z-stacks of apical (A), middle (B), and basal (C) cochlear turns immunostained with a monoclonal antibody against the BK channel (red) and a polyclonal antibody against synapsin (green) to label the efferent presynaptic terminals show that BK channel immunoreactivity in the three rows of outer hair cells (when present) is associated with efferent terminals. (D) Moreover, the size of BK channel immunopuncta increases from apical to middle and basal turns, paralleling an increase in size and likely number of efferent terminals. Data are presented as box plots representing the median (box interior), the 25th and 75th percentile (box boundaries), the 10th and 90th percentile (whiskers), and the 5th and 95th percentile (dots). (E) 3D renderings of confocal z-stacks of a middle cochlear turn immunostained with a polyclonal antibody against the BK channel (red) and a monoclonal antibody against the NaK-ATPase α3 (green) to label the efferent presynaptic terminal membrane shows adjacent but not co localized expression of the BK channel with the presynaptic efferent terminal membrane. The location of the three rows of outer hair cells are indicated (solid arrowheads). Grid dimensions equal 10 µm.

To further explore the association of BK channels with the efferent innervation of OHCs, apical, middle, and basal regions of the organ of Corti were immunolabeled with a monoclonal BK antibody and a polyclonal synapsin antibody to identify efferent terminals. 3D reconstruction of confocal z-stacks (Figure 4A–C) showed that BK channel immunopuncta (if present) were associated with synapsin-labeled efferent terminals. These experiments also enabled a quantitative correlation of efferent innervation with BK channel expression along the tonotopic axis of the rat organ of Corti (Figure 4D). Efferent terminal volume increased from apical (9.5±0.6 µm^3^; n = 138) to middle (22.8±1.1 µm^3^; n = 112) to basal (27.3±1.4 µm^3^; n = 125) regions. In parallel with this gradient in efferent terminal size, there was an increase in the volume of BK immunopuncta from apical (0.3 µm^3^; n = 1 puncta) to middle (9.1±0.4 µm^3^; n = 108 puncta) to basal turns (12.8±0.4 µm^3^; n = 120 puncta) (Figure 4D).

The BK channel immunoreactivity observed in middle and basal OHCs is adjacent to but not precisely overlapping with synapsin immunoreactivity (Figure 4A–C). The lack of BK channel colocalization with the efferent terminal has been reported for other cytoplasmic terminal markers (synaptophysin [13]) and could result from either localization of BK channels to another structure of the presynaptic terminal (on the membrane as opposed to within the cytoplasm by synapsin) or localization of BK channels to the postsynaptic cell (on the OHC membrane). To distinguish these possibilities, the presynaptic efferent terminal membrane was labeled with a monoclonal antibody against the Na,K-ATPase α3 subunit. [34] As shown previously, the Na,K-ATPase α3 subunit is expressed by the efferent terminals but not the Type II afferent fibers contacting the OHCs [34]. 3D reconstructions of confocal z-stacks of midturn regions (Figure 4E) show that the BK channel immunopuncta appear adjacent to but not precisely colocalized with the NaK-ATPase α3-labeled efferent terminal membranes.

**Figure 5:**
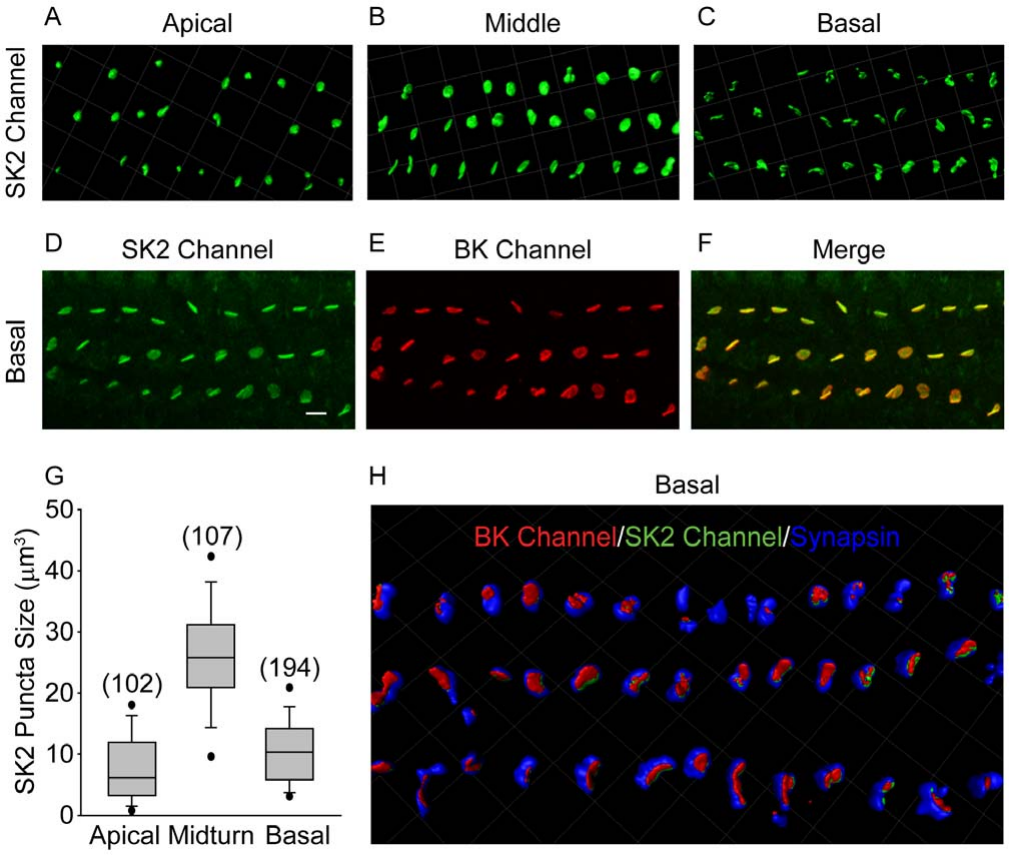
Tonotopic distribution of SK2 channels in outer hair cells. 3D renderings of confocal z-stacks of apical (A), middle (B), and basal (C) cochlear turns immunostained with a polyclonal antibody against the SK2 channel (green) show expression of the SK2 channel in the three rows of outer hair cells in all regions. Confocal z-stacks (23 optical sections) of basal cochlear turns immunostained with a polyclonal antibody against the SK2 channel (green, D) and monoclonal antibody against the BK channel (red, E) show colocalized expression (F) in the three rows of outer hair cells, further suggesting the localization of BK channels to the postsynaptic outer hair cell membrane. (Examination of single optical sections also shows colocalization of the SK2 and BK channel.) The size of the SK2 channel immunopuncta increases from apical to middle turns and then decreases in basal turns. Data are presented as box plots representing the median (box interior), the 25^th^ and 75^th^ percentile (box boundaries), the 10^th^ and 90^th^ percentile (whiskers), and the 5^th^ and 95^th^ percentile (dots). (G) 3D renderings of confocal z-stacks of basal cochlear turns immunostained with a polyclonal antibody against the SK2 channel (green), a monoclonal antibody against the BK channel (red), and a goat polyclonal antibody against synapsin (blue) confirm the colocalized expression of SK2 and BK channels in association with efferent terminals (H) in the three rows of outer hair cells. In basal turns, BK and SK2 channel immunoreactivity are colocalized. Grid dimensions equal 10 µm for top and bottom panels. Scale bar equals 10 µm for middle panels.

Because the expression of the SK2 channel has only been characterized in apical OHCs, SK2 channel expression was quantified in apical, middle, and basal OHCs using immunofluorescence. 3D renderings of confocal z-stacks showed SK2 immunopuncta in OHCs of all three regions (Figure 5A–C). However, in contrast to the increasing size of BK immunopuncta from apical to basal regions, the mean size of SK2 immunopuncta was greatest in middle (26.6±0.9 µm3, n = 107 puncta) compared to apical (7.8±0.5 µm3, n = 102 puncta) and basal regions (10.6±0.4 µm3, n = 192; Figure 5G). Confocal z-stacks of basal turns double labeled with a polyclonal antibody against the SK2 channel (green, Figure 5D) and a monoclonal antibody against the BK channel (Figure 5E) show closely colocalized expression of the two channels (Figure 5F). Colocalized expression of the SK2 and BK channels was confirmed in single optical sections (data not shown). 3D renderings of confocal z-stacks of basal turns immunolabeled with a polyclonal antibody against the SK2 channel (green), a monoclonal antibody against the BK channel (red) and a goat polyclonal antibody against synapsin (blue) also show colocalized expression of the SK2 and BK channels adjacent to but not precisely overlapping with synapsin-labeled efferent terminals (Figure 5H). Thus, SK2 and BK channels are likely both postsynaptic components of efferent-OHC synapses in middle and basal turns of the rat cochlea.

### ACh-evoked K^+^ currents are carried by SK2 channels in apical outer hair cells and BK channels in basal outer hair cells

**Figure 6:**
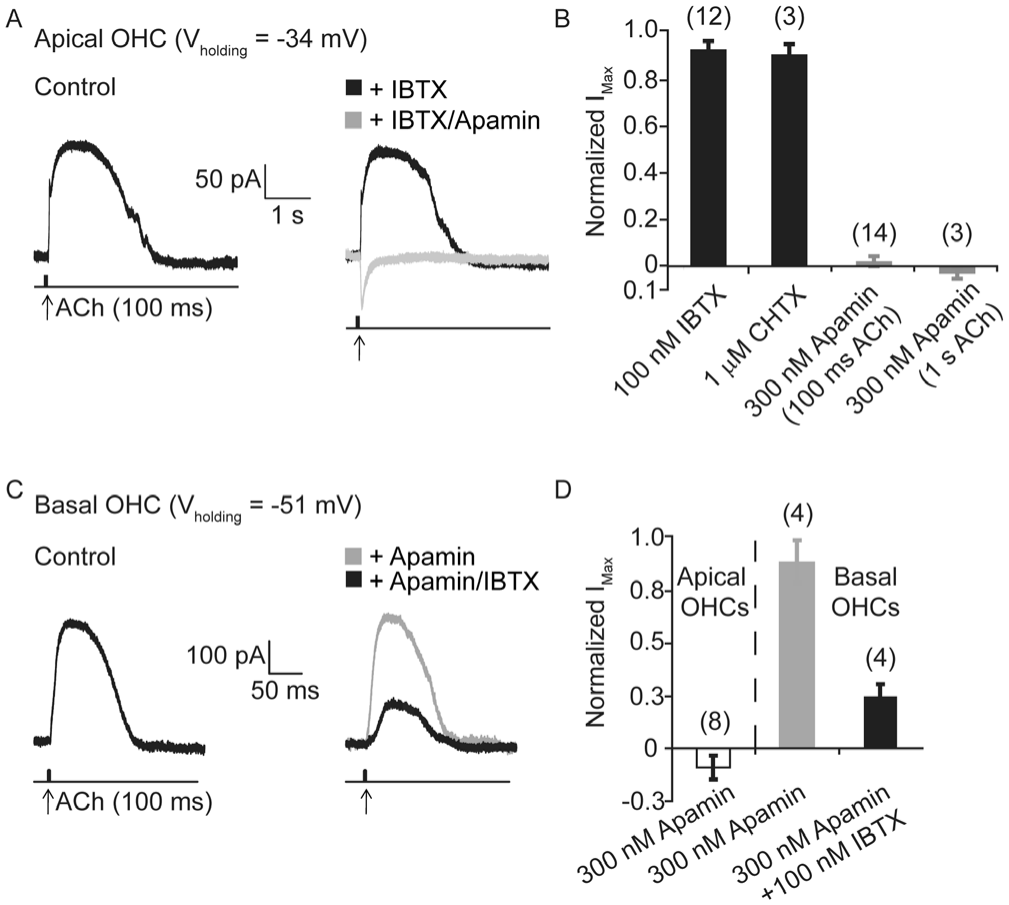
Tonotopic contribution of SK2 and BK channels at the outer hair cell-efferent synapse. (A) Whole-cell currents recorded from an apical outer hair cell (OHC) voltage clamped at −34 mV in response to a short (100 ms) application of 1 mM ACh (time point indicated by arrow) before (control) and after application of either 100 nM IBTX (black) or 300 nM apamin (grey). (B) Bar plot comparing the fractional contribution of IBTX- and CHTX-sensitive BK and apamin-sensitive SK2 currents evoked in response to either 100 ms or 1 s application of ACh show that ACh-evoked currents in apical outer hair cells are predominantly mediated by SK2 channels. (C) Whole-cell currents recorded from a basal outer hair cell (OHC) voltage clamped at −51 mV in response to a short (100 ms) application of 1 mM ACh (timepoint indicated by arrow) before (control) and after application of either 100 nM IBTX (black) or 300 nM apamin (grey). (D) Bar plot comparing the fractional contribution of apamin-sensitive SK2 currents in apical and basal outer hair cells and IBTX-sensitive BK currents in basal outer hair cells in response to application of ACh show that ACh-evoked currents in basal outer hair cells are predominantly mediated by BK channels and not SK2 channels.

The cholinergic inhibition of both immature IHCs and mature OHCs from apical turns is well-characterized and results from an increase in intracellular Ca^2+^ through α9α10-containing ACh receptors and the subsequent activation of small conductance Ca^2+^-activated K^+^ SK2 channels that hyperpolarize the cells [6], [35], [36]. Consistent with these reports, puff application (100 ms) of 1 mM ACh evoked an outward K^+^ current (IK, ACh) in apical OHCs voltage-clamped at −34 mV that was virtually abolished by 300 nM apamin, a specific blocker of SK channels. IK, ACh in control ranged from 60 to 220 pA and was reduced 97.9±2.11% by apamin (n = 14, Figure 6A and B). Increasing the duration of ACh application from 100 ms to 1 s to prolong the activation of AChRs and associated Ca^2+^ influx induced a long lasting IK, ACh that decayed over the course of seconds (data not shown). In this condition, 300 nM apamin also blocked IK, ACh (102±2.56% reduction, n = 3; Figure 6B). Interestingly, IK, ACh recorded from OHCs in middle turns of the cochlea was only partially inhibited with the same concentration of apamin (56.75±9.97%, n = 3, data not shown). Not surprisingly, application of the BK channel blockers IBTX (100 nM) or CHTX (1 µM) only slightly reduced the amplitude and duration of IK, ACh in apical OHCs (Figure 6A and B). Specifically, IK, ACh amplitude and duration were reduced respectively by 8±3.6% and 6.1±4.6% (n = 12, p = 0.0642 and p = 0.1060, respectively) in the presence of IBTX and by 9.9±4.3% and 5.2±3.2% (n = 3) in the presence of CHTX in apical OHCs.

Application of ACh (1 mM, 100 ms) to basal OHCs, voltage-clamped at −51 mV, elicited large, slowly-decaying outward currents generally similar to cholinergically-gated K^+^ currents recorded from apical OHCs. However, the ACh-gated K currents in basal OHCs were relatively insensitive to saturating doses (300 to 600 nM) of apamin (11.1±10% reduction, n = 4, p = 0.1660, Figure 6C and D). In contrast, IK, ACh in apical OHCs was rapidly and fully blocked by apamin at the same holding potential (108.9±5.7% reduction, n = 9; Figure 6D). Instead, basal ACh-gated K currents were substantially blocked (75.1±5.8% reduction, n = 4, p = 0.05) by 100 nM IBTX, a concentration that had no significant effect on apical OHCs. Incubation times in 100 nM IBTX exceeded 4 min and suggest that the residual ACh-evoked currents in basal OHCs may be carried by BK channels that show reduced sensitivity or complete insensitivity to IBTX [37]. Nonetheless, these results show that in basal turns of the cochlea, BK channels contribute more than SK2 channels to IK, ACh and likely the cholinergic efferent inhibition of OHCs.

## Discussion

The objectives of this study were to investigate the tonotopic contribution of BK channels to voltage- and ligand-gated currents in mature OHCs using both whole-cell patch-clamp recordings and immunofluorescence. We found that apical (low frequency) OHCs had no obvious BK immunoreactivity and that voltage-gated K^+^ currents were mainly carried by linopirdine-sensitive (KCNQ4) channels. Similar observations have been made in OHCs from the guinea pig [17], [38] and mouse [4], [10] cochlea. In contrast, basal (high frequency) OHCs showed abundant BK channel immunoreactivity and voltage-gated K^+^ currents carried by BK channels in addition to linopirdine-sensitive current. BK channels in OHCs have been identified in previous studies either by their large unitary conductance [27], [39], [40] or by pharmacology in whole cell recordings [38] but without identification of cochlear location. Others have reported BK channel immunoreactivity in both apical [14] and basal [41] OHCs from the mouse cochlea, but without specific comparison of expression levels.

Immunofluorescence confirmed the expression of BK channels in basal and not apical OHCs and also suggested a tonotopic increase in BK channel expression, with little or no BK channel immunoreactivity observed in apical OHCs and increasing BK channel immunoreactivity observed in middle and basal turns. Additionally, BK immunoreactivity was restricted to the OHC synaptic pole, the site of abundant efferent innervation that originates from the brain. The functional expression of BK currents in basal OHCs (determined by electrophysiology), the lack of colocalization with known presynaptic terminal markers (synapsin and Na,K-ATPase α3), and colocalization with the known postsynaptic terminal protein (SK2 channel) suggest that BK channels in addition to SK2 channels are postsynaptic components of efferent-OHC synapses in middle and basal turns of the rat cochlea. Although immunogold labeling in conjunction with transmission electron microscopy has higher resolution than immunofluorescence and would be the optimal technique to distinguish pre- or postsynapttic localization of BK channels, we found that BK channel immunoreactivity in OHCs was abolished unless organs of Corti were only lightly fixed in paraformaldehyde. Because preparation for electron microscopy requires much harsher fixation that most likely would have abolished BK channel immunoreactivity in the OHCs, we did not perform immunogold experiments to resolve the synaptic localization of BK channels. Importantly, our experiments cannot exclude that there may be an additional population of presynaptic BK channels not detected by our antibodies; in fact, earlier work identified BK channels in isolated cochlear efferent nerve terminals by unitary conductance and pharmacology [42], and transmitter release from efferent terminals on inner hair cells has been shown to be altered by BK channel antagonists [43]. The contribution of SK2 channels to the cholinergic efferent inhibition of apical OHCs is well-characterized. Thus, the colocalized expression of BK and SK2 channels in higher frequency OHCs as well as the observed increase in BK channel immunoreactivity and decrease in SK2 channel immunoreactivity in higher frequency OHCs motivated examination of the tonotopic contribution of BK channels to ligand-gated currents in OHCs.

We found that K^+^ currents elicited by application of ACh were carried exclusively by SK2 channels in apical OHCs and, in marked contrast, by BK channels in basal OHCs. Our findings in apical OHCs are consistent with numerous earlier studies characterizing the SK2-dependent mechanism of synaptic inhibition in both mammalian [6], [22], [35], [36], [44], [45], [46] and non- mammalian hair cells [47]. Because of the technical difficulty of patch clamping OHCs from basal turns, the contribution of K^+^ currents to cholinergic inhibition had not been examined previously in basal OHCs. However, there are reports that ACh-evoked, Ca^2+^-dependent K^+^ currents in vestibular type II hair cells from guinea pig were sensitive to the BK channel blocker IBTX and insensitive to the SK2 channel blocker apamin [48]. Importantly, these hair cells express the same α9α10 nACh receptor subunits found in the cochlea [8], [49]. Hair cell α9α10 nAChRs are characterized by their high Ca^2+^ permeability [50], and their transcript abundance is highest in basal OHCs in the rat cochlea [51], [52]. Given the small size of the postsynaptic space and proximity to synaptoplasmic cisterns [53], activation of α9α10 nACh receptors in basal OHCs could increase the local intracellular [Ca^2+^] to the micromolar range, sufficient to activate colocalized BK channels. Surprisingly, we observed no (or little) effect of apamin on membrane current evoked in basal OHCs by ACh, although immunolabeling shows SK2 channels to be present there. SK2 channels have been shown to be sensitive to voltage-dependent blockade by intracellular divalent cations, including micromolar concentrations of calcium [54]. Perhaps elevated intracellular Ca^2+^ in basal OHCs, due to increased expression of α9α10 nACh receptors, preferentially activates BK channels while blocking SK2 channels in our conditions. This hypothesis suggests future experiments to directly test the effects of Ca^2+^ on voltage-gated BK channels in basal OHCs. Indeed our experiments, with nominal intracellular [Ca^2+^] suggest that BK channels activation by voltage-steps in basal OHCs may occur independently of Ca^2+^ influx through voltage-gated Ca^2+^ channels, as has been reported for BK channels from IHCs [22], [25], [55]. Finally, although not examined electrophysiologically, immunofluorescence revealed relatively equivalent BK and SK2 immunoreactivity in OHCs from middle turns. These results suggest future experiments to determine if ACh-evoked K^+^ currents in OHCs from middle turns are carried by both BK and SK2 channels as suggested by the incomplete block of ACh-evoked currents in middle turns. Such findings would suggest a seamless gradient of ACh-evoked currents carried by exclusively SK2 channels in apical OHCs, a mixture of SK2 and BK channels in middle OHCs, and exclusively BK channels in basal OHCs.

A variety of evidence suggests that K^+^ channels other than the SK2 channel are involved in efferent suppression of high frequency OHCs and that BK channels contribute functionally to basal OHCs. *In vivo* examination of the fast efferent suppression of auditory nerve responses in guinea pigs is dominated by a conductance that is not apamin sensitive [56]. The contribution of BK channels in this work was less clear: a blocker of the BK channel had direct effects on both efferent suppression as well as the amplitude of the auditory nerve responses, most likely reflecting the expression of BK channels in OHCs and IHC afferent dendrites. The functional contribution of BK channels to high frequency OHCs is suggested by observations of knock out mice lacking either the BK or SK2 channels. In a strain of mice predisposed to age related hearing loss, absence of the BK channel resulted in accelerated high frequency hearing loss that was attributed to the loss of high frequency (basal) OHCs [13]. Although the mechanism by which the absence of the BK channel contributes to OHC degeneration is unknown, perhaps loss of the protective effects of efferent inhibition supported by BK channels in basal OHCs increases their susceptibility to loss and accelerates subsequent high frequency hearing loss. Examination of mice lacking the SK2 channel show degeneration of efferent-OHC synapses, suggesting the requirement of SK2 channels to maintain efferent innervation of OHCs [9], [57]. Interestingly, loss of efferent innervation in basal OHCs is slower and persists for several weeks [57]. The additional expression of BK channels in basal efferent-OHC synapses may protect the survival of these synapses in an activity-dependent manner.

Much of our knowledge of the K^+^ channels supporting cholinergic inhibition of hair cells comes from investigations of the efferent-hair cell synapses from non mammalian preparations [47], the transient efferent-IHC synapses found before the onset of hearing [35], [36], [58] and apical turn efferent-OHC synapses [6]. In these preparations, the inhibitory K^+^ current is unequivocally and exclusively mediated by the SK2 channel. Our findings that BK channels can carry this inhibitory current in basal OHCs suggest that efferent synapses may employ fundamentally different mechanisms of action to inhibit high frequency OHCs. BK channels may mediate inhibition exclusive of SK2 channels or, as suggested by the colocalization of BK and SK2 channels, both channels may contribute to efferent inhibition depending upon the degree of presynaptic activity. Specifically, the high Ca^2+^ affinity of SK2 channels suggests they may be activated by low or moderate efferent activity, whereas BK channels, with lower Ca^2+^ affinity, would be preferentially activated by stronger efferent activity (as suggested by our experiments with long puff applications of ACh). Ultimately, both the BK and SK2 channels may be required to modulate the strength of postsynaptic inhibition.

Molecules empowering the remarkable sensitivity and frequency selectivity of mammalian hearing, such as the α10 nAChR subunit and the electromotor protein prestin, have undergone adaptive evolution in the mammalian lineage [59]. Recruitment of the BK channel to efferent inhibition may be yet another adaptation to support mammalian hearing. Future experiments examining the contribution of BK and SK2 channels to high frequency efferent inhibition *in vitro* as well as efferent suppression *in vivo* will be essential to uncovering the role of BK channels in efferent inhibition of the cochlea.

## Methods

### Animal procedures and tissue preparation

Electrophysiology and immunofluorescence experiments were performed using semi-intact organs of Corti dissected from the cochleae of hearing Sprague-Dawley rats aged postnatal days (P) 19 to 24. All animal protocols were approved by the Johns Hopkins University (RA08M198) and the University of North Carolina at Wilmington (A0809-025) Institutional Animal Care and Use Committees. Rats were overdosed with an inhalant anesthetic (Isoflurane, USF, MINRAD Inc, Bethlehem, USA) and killed by decapitation. The cochleae were dissected in either external solution maintained at 4°C and containing (in mM): 5.8 KCl; 144 NaCl; 0.9 MgCl2; 1.3 CaCl2; 0.7 NaH2PO4; 10 HEPES and 5 glucose (pH 7.4, ∼300 mOsm) for electrophysiological recordings or phosphate buffered saline maintained at 4°C for immunofluorescence experiments. For each cochlea, the bone was entirely removed to expose the organ of Corti. Two tonotopic regions of the organ of Corti were examined: the apex (or low frequency region) and base (or high frequency region). For electrophysiology, apical and basal sections were isolated together and the tectorial membrane was not removed to prevent detachment of the basal OHCs. Nevertheless, the third row usually became detached during the dissection. Approximately one-third of the dissections gave a usable preparation. A recording was obtained from about half of these preparations. To correlate the localization of BK channels with their function in each region, identical regions (shown in Figure 1A) were used for both electrophysiology and immunofluorescence experiments.

### Electrophysiology

Whole cell, tight seal voltage-clamp recordings from all rows of OHCs were made within 1 hour after the dissection of the organ of Corti. OHCs from basal turns were extremely fragile, with recording times generally lasting fewer than 20 min. Unless specified in the text, the composition of the external solution was the same as the solution used for dissecting the cochlea. In some experiments the [Ca^2+^] of the external solution was varied. The free [Ca^2+^] was calculated using WEBMAXC v2.22 (http://stanford.edu/~cpatton/max.html). The internal solution (in the recording pipette) contained (in mM): 120 Kgluconate; 20 KCl; 5 EGTA; 5 HEPES; 2.5 Na2ATP; 0.1 Ca^2+^; 3.5 MgCl2; and 10 Na-Phosphocreatine (pH 7.2, 285 mOsm). In some experiments, the concentration of EGTA in the internal solution was reduced to 0.1 mM. Currents were recorded at room temperature (22 to 25°C) using PClamp 10.2 software and a Multiclamp 700B amplifier (Axon Instruments, Sunnyvale, CA). Data were digitized at 10 to 20 KHz and low pass filtered at 5 to 10 KHz with a Digidata 1440A and analyzed offline using Origin software (OriginLab, Northampton, MA, USA). Series resistances (typically 7 to 15 MΩ) were compensated up to 50% on-line. The steady state holding potentials are corrected for a measured 11 mV liquid junction potential and the uncompensated series resistance.

### Drug application

Iberiotoxin (IBTX), charybdotoxin (CHTX), paxilline, XE991, and linopirdine were purchased from Tocris Bioscience (Ellisville, MO, USA). Apamin was purchased from Sigma-Aldrich (St Louis, MO). External solutions containing toxins were added to the preparation by a gravity-fed perfusion system (Warner Instruments, Hamden, CT) at a rate of 2 to 3 mL/min with perfusion and suction pipettes placed in opposite sides of the recording chamber. Responses to toxin applications were recorded after at least 3 min of perfusion, sufficient time to allow complete exchange of solution in the recording chamber. Blocking effects of apamin and IBTX stocks were tested, respectively, in apical OHCs and apical IHCs, with currents completely blocked within 1 min. In some cells, even longer applications (5 to 6 min) of higher concentrations of apamin (600 nM) were tested.

To activate the α9α10-containing nACh receptors, 1 mM acetylcholine (ACh) was delivered to the OHCs from a pipette positioned ∼50 µm from the cell using positive pressure (2 to 4 psi) delivered through a computer-controlled valve (Picospritzer, Parker Hannifin, Pine Brook, NJ).

### Statistics

All means are reported ± s. e. m. in both text and figures. A normality test was performed to verify the Gaussian distribution of the data prior to performing a one-tailed (paired) Student's t test (using an α level set to 0.05). Statistical analyses were performed using Graphpad Prism4 (Graphpad Inc., La Jolla, CA, USA). Box plots were generated in SigmaPlot 10 (Systat Software, Inc., San Jose, CA, USA).

### Immunofluorescence

Immunofluorescent staining was performed as described previously [34] using the following antibodies: mouse monoclonal anti-BK (L6/23; 1∶500; provided by Dr. James Trimmer, UC Davis, CA), rabbit polyclonal anti BK (APC021; 1∶500; Alomone Labs, Jerusalem, Israel), rabbit polyclonal anti-SK2 (1∶500; provided by Dr John Adelman, Vollum Institute, University of Oregon Health Sciences), goat polyclonal anti-prestin (1∶300; Santa Cruz Biotechnology, Santa Cruz, CA), rabbit polyclonal anti-synapsin (AB1543P; 1∶600; Chemicon/Millipore, Temecula, CA); and mouse monoclonal anti-Na-KATPase α3 subunit (MA3-915; 1∶600; Affinity Bioreagents, Rockford, IL). Secondary antibodies (Alexa Fluor 488, 594, and 633 generated in either goat or donkey) were purchased from Molecular Probes/Invitrogen (Carlsbad, CA) and diluted 1∶1000 in blocking buffer.

### Verification of antibody specificity

Specificity of the recently generated monoclonal antibody against the BK channel (L6/23) was verified by western blot analysis. The L6/23 antibody detects bands of the predicted molecular weight in western blots of rat and wildtype mouse brain membrane preparations and detects no bands in blots of brain membrane preparations from BK channel (α subunit) knockout mice (Figure S2A). Specificity of the polyclonal antibody against the BK channel (APC021) has been verified by the vendor using western blot analysis of rat brain membrane preparations and immunofluorescence to show membrane localization in cells of the rat interpeduncular nucleus. Additionally, the monoclonal (L6/23) and polyclonal (APC021) BK channel antibodies show colocalized immunoreactivity in both the inner and outer hair cells from midbasal turns of the rat organ of Corti (Figure S2B). Specificity of the polyclonal SK2 channel antibody (generated by Dr. John Adelman, Vollum Institute, University of Oregon Health Sciences) has been previously described [60] and was additionally verified by western blot analysis using brain membrane preparations from wild type and SK2 channel knockout mice (personal communication from Dr. Chris Bond, Vollum Institute, University of Oregon Health Sciences).

### Microscopy and image analysis

Fluorescent images were acquired using an Olympus Fluoview FV1000 confocal microscope with a 60X Olympus PlanoApo oil immersion lens (N. A. 1.42) under the control of the Olympus Fluoview FV1000 version 1.6a software (Olympus Corporation, Center Valley, PA, USA). Z-stacks (6 to 90 optical sections) were collected at 0.3 to 0.5 µm intervals. Images are presented as z-projections through the entire optical stack unless otherwise noted. 3D renderings of confocal z-stacks were generated and analyzed using Imaris 6.4 3D image visualization and analysis software (Bitplane Inc., Saint Paul, MN, USA). Confocal z-stacks were opened and resampled to contain the region of interest. Contour surfaces were made for each channel using a surface area detail of 0.2 µm, manually adjusting the absolute intensity and, in some cases, manually splitting touching objects. Volumes of surfaces (µm^3^) were determined using the statistics option in Imaris and exported to Excel (Microsoft) for subsequent analysis.

## Supporting Information

Figure S1 KCNQ4 but not BK channels contribute to voltage-gated K^+^ currents in apical outer hair cells. (A) Bar plot comparing the fractional contribution of IBTX-, paxilline-, XE991, and linopirdine-sensitive currents to apical outer hair cells at 49 mV show that apical outer hair cells express predominantly XE991- and linopirdine-sensitive KCNQ4 currents and little or no BK currents. (B) Membrane currents recorded from an apical outer hair cell before (control, black trace), after bath application of extracellular solution containing elevated Ca^2+^ (10 mM Ca^2+^, grey trace), and after bath application of elevated Ca^2+^ and 100 nM IBTX (10 mM Ca^2+^ + IBTX, light grey trace). (C). Bar plot comparing the mean current density at 49 mV in the experimental conditions shown in B confirm the absence of IBTX-sensitive currents in apical outer hair cells even in the presence of elevated extracellular Ca^2+^.
(3.19 MB TIF)

Figure S2 Specificity of the monoclonal and polyclonal BK channel antibodies. Specificity of the monoclonal antibody against the BK channel (L6/23) was verified by western blot analysis. The L6/23 antibody detects bands of the predicted molecular weight in western blots of rat and wild type mouse brain membrane preparations and detects no bands in blots of brain membrane preparations from BK channel (α subunit) knockout mice (A). Additionally, the monoclonal (L6/23) and polyclonal (APC021) BK channel antibodies show co localized immunoreactivity in both the single row of inner hair cells and three rows of outer hair cells from midbasal turn of the rat organ of Corti (B).
(4.57 MB TIF)

## References

[1] Dallos P, Harris D (1978). Properties of auditory nerve responses in absence of outer hair cells.. J Neurophysiol.

[2] Ryan A, Dallos P (1975). Effect of absence of cochlear outer hair cells on behavioural auditory threshold.. Nature.

[3] Dallos P, Cheatham MA (1992). Cochlear hair cell function reflected in intracellular recordings in vivo.. Soc Gen Physiol Ser.

[4] Marcotti W, Kros CJ (1999). Developmental expression of the potassium current IK,n contributes to maturation of mouse outer hair cells.. J Physiol.

[5] Nakagawa T, Kakehata S, Akaike N, Komune S, Takasaka T (1994). Voltage-dependent channels in dissociated outer hair cells of the guinea pig.. Eur Arch Otorhinolaryngol.

[6] Oliver D, Klocker N, Schuck J, Baukrowitz T, Ruppersberg JP (2000). Gating of Ca2+-activated K+ channels controls fast inhibitory synaptic transmission at auditory outer hair cells.. Neuron.

[7] Elgoyhen AB, Johnson DS, Boulter J, Vetter DE, Heinemann S (1994). Alpha 9: an acetylcholine receptor with novel pharmacological properties expressed in rat cochlear hair cells.. Cell.

[8] Elgoyhen AB, Vetter DE, Katz E, Rothlin CV, Heinemann SF (2001). alpha10: a determinant of nicotinic cholinergic receptor function in mammalian vestibular and cochlear mechanosensory hair cells.. Proc Natl Acad Sci U S A.

[9] Kong JH, Adelman JP, Fuchs PA (2008). Expression of the SK2 calcium-activated potassium channel is required for cholinergic function in mouse cochlear hair cells.. J Physiol.

[10] Kharkovets T, Dedek K, Maier H, Schweizer M, Khimich D (2006). Mice with altered KCNQ4 K+ channels implicate sensory outer hair cells in human progressive deafness.. EMBO J.

[11] Kharkovets T, Hardelin JP, Safieddine S, Schweizer M, El-Amraoui A (2000). KCNQ4, a K+ channel mutated in a form of dominant deafness, is expressed in the inner ear and the central auditory pathway.. Proc Natl Acad Sci U S A.

[12] Pyott SJ, Meredith AL, Fodor AA, Vazquez AE, Yamoah EN (2007). Cochlear function in mice lacking the BK channel alpha, beta1, or beta4 subunits.. J Biol Chem.

[13] Ruttiger L, Sausbier M, Zimmermann U, Winter H, Braig C (2004). Deletion of the Ca2+-activated potassium (BK) alpha-subunit but not the BKbeta1-subunit leads to progressive hearing loss.. Proc Natl Acad Sci U S A.

[14] Engel J, Braig C, Ruttiger L, Kuhn S, Zimmermann U (2006). Two classes of outer hair cells along the tonotopic axis of the cochlea.. Neuroscience.

[15] Langer P, Grunder S, Rusch A (2003). Expression of Ca2+-activated BK channel mRNA and its splice variants in the rat cochlea.. J Comp Neurol.

[16] Mammano F, Ashmore JF (1996). Differential expression of outer hair cell potassium currents in the isolated cochlea of the guinea-pig.. J Physiol.

[17] Housley GD, Ashmore JF (1992). Ionic currents of outer hair cells isolated from the guinea-pig cochlea.. J Physiol.

[18] Latorre R, Brauchi S (2006). Large conductance Ca2+-activated K+ (BK) channel: activation by Ca2+ and voltage.. Biol Res.

[19] Marty A (1981). Ca-dependent K channels with large unitary conductance in chromaffin cell membranes.. Nature.

[20] Pallotta BS, Magleby KL, Barrett JN (1981). Single channel recordings of Ca2+-activated K+ currents in rat muscle cell culture.. Nature.

[21] Muller M (1991). Frequency representation in the rat cochlea.. Hear Res.

[22] Marcotti W, Johnson SL, Kros CJ (2004). Effects of intracellular stores and extracellular Ca(2+) on Ca(2+)-activated K(+) currents in mature mouse inner hair cells.. J Physiol.

[23] Oliver D, Taberner AM, Thurm H, Sausbier M, Arntz C (2006). The role of BKCa channels in electrical signal encoding in the mammalian auditory periphery.. J Neurosci.

[24] Pyott SJ, Glowatzki E, Trimmer JS, Aldrich RW (2004). Extrasynaptic localization of inactivating calcium-activated potassium channels in mouse inner hair cells.. J Neurosci.

[25] Thurm H, Fakler B, Oliver D (2005). Ca2+-independent activation of BKCa channels at negative potentials in mammalian inner hair cells.. J Physiol.

[26] Spreadbury IC, Kros CJ, Meech RW (2004). Effects of trypsin on large-conductance Ca(2+)-activated K(+) channels of guinea-pig outer hair cells.. Hear Res.

[27] van Den Abbeele T, Teulon J, Huy PT (1999). Two types of voltage-dependent potassium channels in outer hair cells from the guinea pig cochlea.. Am J Physiol.

[28] Sanchez M, McManus OB (1996). Paxilline inhibition of the alpha-subunit of the high-conductance calcium-activated potassium channel.. Neuropharmacology.

[29] Kubisch C, Schroeder BC, Friedrich T, Lutjohann B, El-Amraoui A (1999). KCNQ4, a novel potassium channel expressed in sensory outer hair cells, is mutated in dominant deafness.. Cell.

[30] Cui J, Yang H, Lee US (2009). Molecular mechanisms of BK channel activation.. Cell Mol Life Sci.

[31] Knirsch M, Brandt N, Braig C, Kuhn S, Hirt B (2007). Persistence of Ca(v)1.3 Ca2+ channels in mature outer hair cells supports outer hair cell afferent signaling.. J Neurosci.

[32] Hafidi A, Beurg M, Dulon D (2005). Localization and developmental expression of BK channels in mammalian cochlear hair cells.. Neuroscience.

[33] Belyantseva IA, Adler HJ, Curi R, Frolenkov GI, Kachar B (2000). Expression and localization of prestin and the sugar transporter GLUT-5 during development of electromotility in cochlear outer hair cells.. J Neurosci.

[34] McLean WJ, Smith KA, Glowatzki E, Pyott SJ (2009). Distribution of the Na,K-ATPase alpha subunit in the rat spiral ganglion and organ of corti.. J Assoc Res Otolaryngol.

[35] Glowatzki E, Fuchs PA (2000). Cholinergic synaptic inhibition of inner hair cells in the neonatal mammalian cochlea.. Science.

[36] Katz E, Elgoyhen AB, Gomez-Casati ME, Knipper M, Vetter DE (2004). Developmental regulation of nicotinic synapses on cochlear inner hair cells.. J Neurosci.

[37] Meera P, Wallner M, Toro L (2000). A neuronal beta subunit (KCNMB4) makes the large conductance, voltage- and Ca2+-activated K+ channel resistant to charybdotoxin and iberiotoxin.. Proc Natl Acad Sci U S A.

[38] Nenov AP, Norris C, Bobbin RP (1997). Outwardly rectifying currents in guinea pig outer hair cells.. Hear Res.

[39] Ashmore JF, Meech RW (1986). Ionic basis of membrane potential in outer hair cells of guinea pig cochlea.. Nature.

[40] Gitter AH, Fromter E, Zenner HP (1992). C-type potassium channels in the lateral cell membrane of guinea-pig outer hair cells.. Hear Res.

[41] Winter H, Braig C, Zimmermann U, Engel J, Rohbock K (2007). Thyroid hormone receptor alpha1 is a critical regulator for the expression of ion channels during final differentiation of outer hair cells.. Histochem Cell Biol.

[42] Wangemann P, Takeuchi S (1993). Maxi-K+ channel in single isolated cochlear efferent nerve terminals.. Hear Res.

[43] Zorrilla de San Martin J, Pyott S, Ballestero J, Katz E (2010). Ca(2+) and Ca(2+)-activated K(+) channels that support and modulate transmitter release at the olivocochlear efferent-inner hair cell synapse.. J Neurosci.

[44] Dulon D, Luo L, Zhang C, Ryan AF (1998). Expression of small-conductance calcium-activated potassium channels (SK) in outer hair cells of the rat cochlea.. Eur J Neurosci.

[45] Marcotti W, Johnson SL, Kros CJ (2004). A transiently expressed SK current sustains and modulates action potential activity in immature mouse inner hair cells.. J Physiol.

[46] Nenov AP, Norris C, Bobbin RP (1996). Acetylcholine response in guinea pig outer hair cells. II. Activation of a small conductance Ca(2+)-activated K+ channel.. Hear Res.

[47] Yuhas WA, Fuchs PA (1999). Apamin-sensitive, small-conductance, calcium-activated potassium channels mediate cholinergic inhibition of chick auditory hair cells.. J Comp Physiol A.

[48] Kong WJ, Guo CK, Zhang S, Hao J, Wang YJ (2005). The properties of ACh-induced BK currents in guinea pig type II vestibular hair cells.. Hear Res.

[49] Hiel H, Elgoyhen AB, Drescher DG, Morley BJ (1996). Expression of nicotinic acetylcholine receptor mRNA in the adult rat peripheral vestibular system.. Brain Res.

[50] Weisstaub N, Vetter DE, Elgoyhen AB, Katz E (2002). The alpha9alpha10 nicotinic acetylcholine receptor is permeable to and is modulated by divalent cations.. Hear Res.

[51] Morley BJ, Simmons DD (2002). Developmental mRNA expression of the alpha10 nicotinic acetylcholine receptor subunit in the rat cochlea.. Brain Res Dev Brain Res.

[52] Simmons DD, Morley BJ (1998). Differential expression of the alpha 9 nicotinic acetylcholine receptor subunit in neonatal and adult cochlear hair cells.. Brain Res Mol Brain Res.

[53] Lioudyno M, Hiel H, Kong JH, Katz E, Waldman E (2004). A “ynaptoplasmic cistern”mediates rapid inhibition of cochlear hair cells.. J Neurosci.

[54] Soh H, Park CS (2002). Localization of divalent cation-binding site in the pore of a small conductance Ca(2+)-activated K(+) channel and its role in determining current-voltage relationship.. Biophys J.

[55] Kros CJ, Crawford AC (1990). Potassium currents in inner hair cells isolated from the guinea-pig cochlea.. J Physiol.

[56] Yoshida N, Liberman MC, Brown MC, Sewell WF (2001). Fast, but not slow, effects of olivocochlear activation are resistant to apamin.. J Neurophysiol.

[57] Murthy V, Maison SF, Taranda J, Haque N, Bond CT (2009). SK2 channels are required for function and long-term survival of efferent synapses on mammalian outer hair cells.. Mol Cell Neurosci.

[58] Simmons DD, Moulding HD, Zee D (1996). Olivocochlear innervation of inner and outer hair cells during postnatal maturation: an immunocytochemical study.. Brain Res Dev Brain Res.

[59] Franchini LF, Elgoyhen AB (2006). Adaptive evolution in mammalian proteins involved in cochlear outer hair cell electromotility.. Mol Phylogenet Evol.

[60] Lin MT, Lujan R, Watanabe M, Adelman JP, Maylie J (2008). SK2 channel plasticity contributes to LTP at Schaffer collateral-CA1 synapses.. Nat Neurosci.

