# Supplementary figures and images for: BK Channels Mediate Cholinergic Inhibition of High Frequency Cochlear Hair Cells
Source: PLoS One. 2010 Nov 4;5(11):e13836. doi: 10.1371/journal.pone.0013836 (PMC2973960; doi:10.1371/journal.pone.0013836)

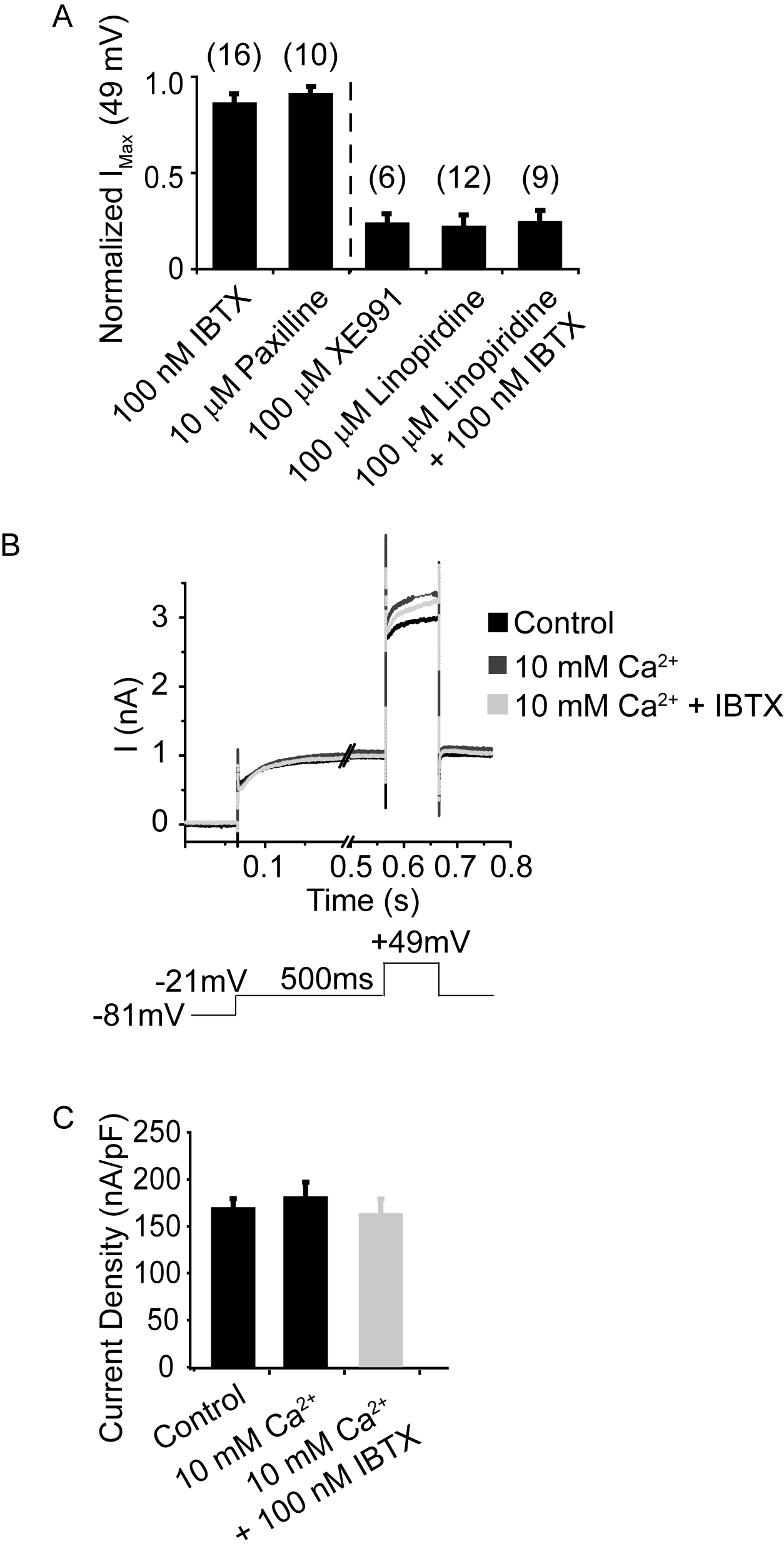

Supplement: Figure S1 — KCNQ4 but not BK channels contribute to voltage-gated K+ currents in apical outer hair cells. (A) Bar plot comparing the fractional contribution of IBTX-, paxilline-, XE991, and linopirdine-sensitive currents to apical outer hair cells at 49 mV show that apical outer hair cells express predominantly XE991- and linopirdine-sensitive KCNQ4 currents and little or no BK currents. (B) Membrane currents recorded from an apical outer hair cell before (control, black trace), after bath application of extracellular solution containing elevated Ca2+ (10 mM Ca2+, grey trace), and after bath application of elevated Ca2+ and 100 nM IBTX (10 mM Ca2+ + IBTX, light grey trace). (C). Bar plot comparing the mean current density at 49 mV in the experimental conditions shown in B confirm the absence of IBTX-sensitive currents in apical outer hair cells even in the presence of elevated extracellular Ca2+. (3.19 MB TIF) [file pone.0013836.s001.tif]

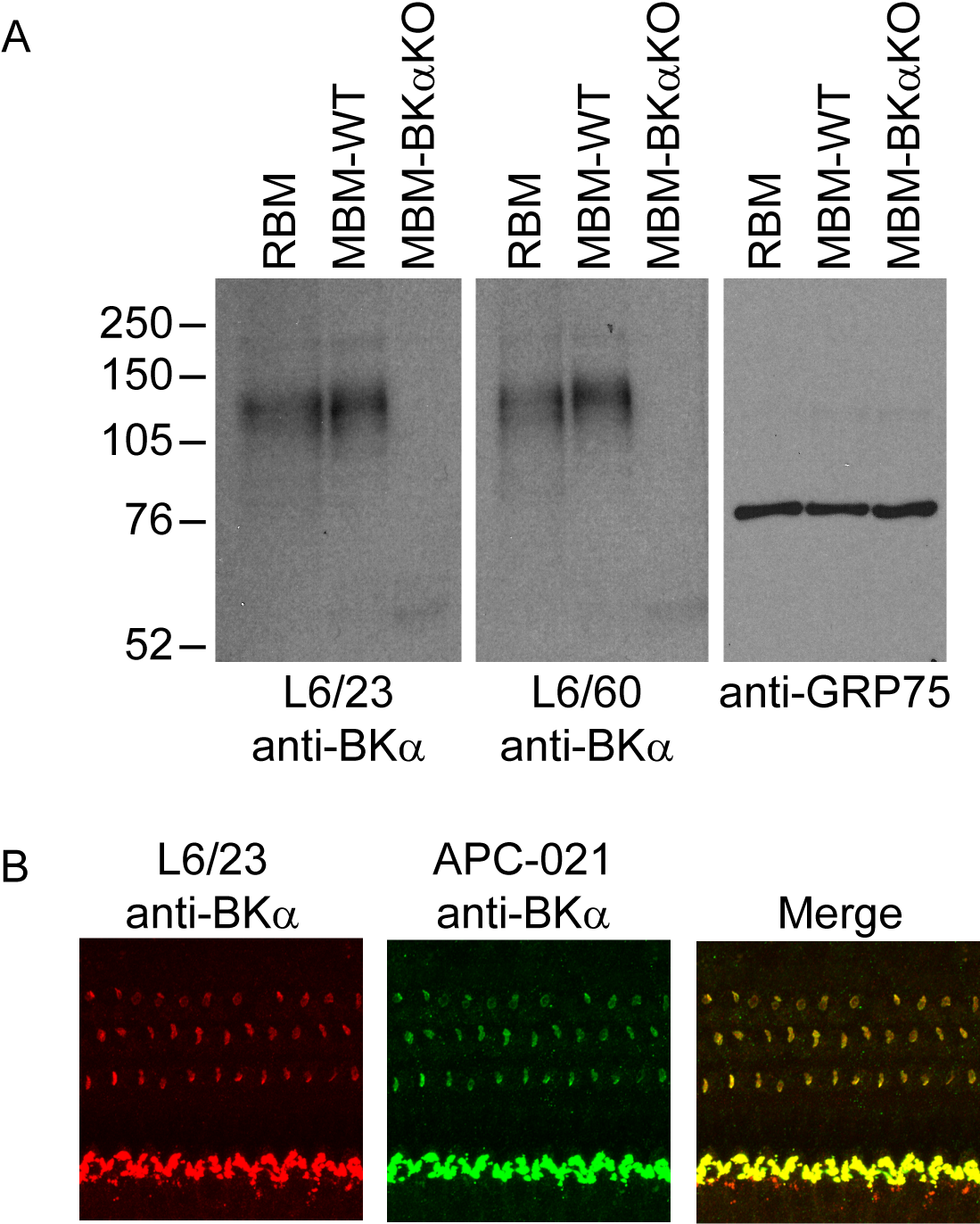

Supplement: Figure S2 — Specificity of the monoclonal and polyclonal BK channel antibodies. Specificity of the monoclonal antibody against the BK channel (L6/23) was verified by western blot analysis. The L6/23 antibody detects bands of the predicted molecular weight in western blots of rat and wild type mouse brain membrane preparations and detects no bands in blots of brain membrane preparations from BK channel (α subunit) knockout mice (A). Additionally, the monoclonal (L6/23) and polyclonal (APC021) BK channel antibodies show co localized immunoreactivity in both the single row of inner hair cells and three rows of outer hair cells from midbasal turn of the rat organ of Corti (B). (4.57 MB TIF) [file pone.0013836.s002.tif]
